# Identification of the Key MicroRNAs and the miRNA-mRNA Regulatory Pathways in Prostate Cancer by Bioinformatics Methods

**DOI:** 10.1155/2018/6204128

**Published:** 2018-06-20

**Authors:** Dongyang Li, Xuanyu Hao, Yongsheng Song

**Affiliations:** ^1^Department of Urology, Shengjing Hospital of China Medical University, Shenyang, Liaoning 110004, China; ^2^Department of Rheumatology and Immunology, Shengjing Hospital of China Medical University, Shenyang, Liaoning 110022, China

## Abstract

**Objective:**

To identify key microRNAs (miRNAs) and their regulatory networks in prostate cancer.

**Methods:**

Four miRNA and three gene expression microarray datasets were downloaded for analysis from Gene Expression Omnibus database. The differentially expressed miRNA and genes were accessed by a GEO2R. Functional and pathway enrichment analyses were performed using the DAVID program. Protein-protein interaction (PPI) and miRNA-mRNA regulatory networks were constructed using the STRING and Cytoscape tool. Moreover, the results and clinical significance were validated in TCGA data.

**Results:**

We identified 26 significant DEMs, 633 upregulated DEGs, and 261 downregulated DEGs. Functional enrichment analysis indicated that significant DEGs were related to TGF-beta signaling pathway and TNF signaling pathway in PCa. Key DEGs such as HSPA8, PPP2R1A, CTNNB1, ADCY5, ANXA1, and COL9A2 were found as hub genes in PPI networks. TCGA data supported our results and the miRNAs were correlated with clinical stages and overall survival.

**Conclusions:**

We identified 26 miRNAs that may take part in key pathways like TGF-beta and TNF pathways in prostate cancer regulatory networks. MicroRNAs like miR-23b, miR-95, miR-143, and miR-183 can be utilized in assisting the diagnosis and prognosis of prostate cancer as biomarkers. Further experimental studies are required to validate our results.

## 1. Introduction

Prostate cancer (PCa) is a major cancer bothering men worldwide. With an estimated 19% new cancer incidence in men, PCa continues to hold the highest morbidity among all cancer types in the United States [1]. During the past decades, urologists have made great progress in the diagnosis and treatment of PCa [2]. Currently, the new types of biomarkers such as liquid biopsy and imaging biomarker start to make contributions to clinical decision making [3–5]. However, PCa is a heterogeneous cancer with individual disparity; therefore, genomic and molecular resources still play an important role in discriminating aggressive cases and aiding prognosis prediction [6].

MicroRNA (miRNA), also known as short noncoding RNA, is a group of single-stranded RNA molecules that act as posttranscriptional gene regulation [7]. MiRNAs are involved in many cellular biological processes such as proliferation, migration, apoptosis, invasion, and epithelial-mesenchymal transition (EMT). Accumulating evidence shows that miRNAs can functionally act as oncogene or tumor suppressors [8]. In addition, circulating miRNA can be detected without invasion like biopsy [9]. Hence, miRNAs can be applied to help diagnosis and prognosis. Multiple studies of miRNA-PCa have been performed, but they often had the disadvantages of heterogeneous design, small sample size, and less clinical information.

To the best of our knowledge, there are few studies integrating microarray datasets to access key molecules and investigate miRNA-mRNA regulatory networks. The objective of this study is to find the key miRNAs and their potential regulatory mechanisms in PCa by bioinformatic approaches.

## 2. Materials and Methods

### 2.1. Microarray Data

Three gene expression profiles (GSE55945, GSE60329, and GSE103512) and four miRNA expression profiles (GSE26367, GSE64333, GSE21036, and GSE54516) were obtained from the Gene Expression Omnibus (GEO, http://www.ncbi.nlm.nih.gov/geo). The profiles based on PCa cell lines or xenograft models were excluded; thus, only PCa patients and healthy controls were preserved in this study. Every included dataset contains more than ten PCa samples and normal prostate tissues. The characteristics of these profiles are shown in Table 1.

### 2.2. Data Processing

We performed the comparison on the two groups of samples (PCa versus normal prostate tissues) in each GEO dataset to identify differentially expressed genes (DEGs) and differentially expressed miRNAs (DEMs). The comparison was launched by a limma R package based online program, GEO2R (http://www.ncbi.nlm.nih.gov/geo/geo2r/). The adjusted *P* value (adj. *P* value) from the Benjamini–Hochberg method could correct the false positive results. So we chose the “adj. *P* value<0.05” and “|logFC|>1” as a primary cut-off criteria to interpret the results. Among the DEMs from the four datasets, only those appeared in two or more datasets were considered as the significant DEMs.

### 2.3. Identification of miRNA Targets

The MiRWalk 2.0 database provides a large collection of predicted and experimentally verified miRNAs-targets binding sites information [10]. We downloaded the significant DEMs-mRNA intersection data from the MiRWalk 2.0-validated-target miRNA-gene retrieval system, which contains all literature-reported miRNA-target genes. However, the validated target genes were from various diseases models, so the expression of those genes in PCa might not be consistent with other diseases. We subsequently selected the genes from the intersection between the significant DEMs-mRNA intersection data and the DEGs from the three GEO datasets. These genes were defined as the significant DEGs.

### 2.4. Functional Enrichment Analysis

The Database for Annotation, Visualization, and Integrated Discovery (DAVID) is an online program that offers functional annotation of enormous quantity of genes derived from various genomic resources [11]. We used the DAVID database to perform Gene Ontology (GO) and Kyoto Encyclopedia of Genes and Genomes (KEGG) pathway analysis on significant DEGs. The species was limited to* Homo sapiens* and the “*P* value <0.05” was considered statistically significant.

### 2.5. Interaction and Regulatory Network Establishment

Search Tool for the Retrieval of Interacting Genes/Proteins (STRING) was used to construct protein-protein interaction (PPI) network. The STRING database collects and predicts interaction information from genomic context predictions, high-throughput lab experiments, coexpression, automated text-mining, and previous knowledge in databases [12]. To access more objective and reliable results, we restricted the sources as high-throughput lab experiments and previous knowledge in databases. In addition, the minimum interaction score was set at high confidence (0.700).

The miRTarBase (http://mirtarbase.mbc.nctu.edu.tw/) is a miRNA intersection database based on validation from experiments such as assay, microarray, sequencing quantitative PCR, and western blot. The miRanda algorithm from the “microRNA.org-Targets and Expression” was freely available and can be applied to the whole genome sequences using identified miRNA sequences [13]. The two programs above were used to predict target mRNAs of the significant DEMs. When two miRNAs shared a common target mRNA; they might exist in a similar regulatory pathway. The miRNA-mRNA regulatory network was visualized by Cytoscape 3.6.0 [14].

### 2.6. Validation and Survival Analysis

The Cancer Genome Atlas (TCGA) database provides abundant clinical information from huge sample size. Therefore, the dataset “TCGA-PRAD” was used to find if the significant DEMs were correlated with survival outcome. The dataset “TCGA-PRAD” consists of 498 PCa samples with 52 normal prostate samples. Among the 494 clinical outcome events available, 484 patients were living and 10 were dead when the follow-up ended. We examined the significant DEMs expression, relationship with PCa stages, and survival. The “t-test” was applied to check expression level, while the chi-square test was used on different stages. The Kaplan-Meier plots were constructed by OncomiR [15]. The “*P *value <0.05” was considered statistically significant.

## 3. Results

### 3.1. DEGs/DEMs Identification

**Figure 1:**
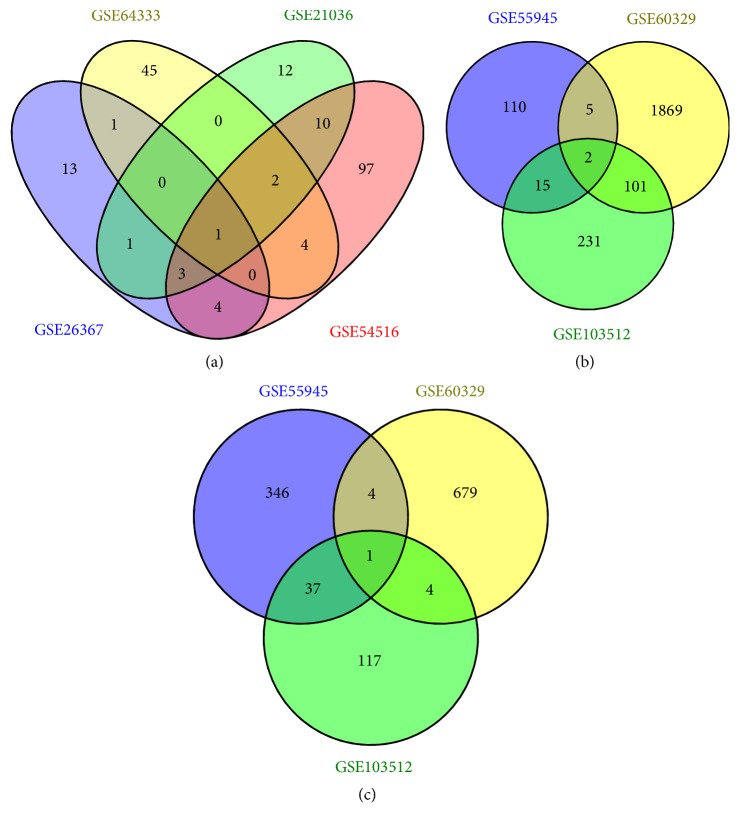
Venn diagram of DEM/DEG selection in different datasets. (a) DEMs; (b) upregulated DEGs; and (c) downregulated DEGs.

After being compared with normal prostate tissues, the results of GEO2R analysis showed that there were totally 2333 upregulated DEGs (Figure 1(b)), 1188 downregulated DEGs (Figure 1(c)), and 193 DEMs (Figure 1(a)) in PCa samples. Among them, 26 DEMs appeared in the results of two datasets at least, so they were identified as significant DEMs. Additionally, miR-183 was the only DEM in all four datasets simultaneously. The following DEMs were found in three datasets: miR-96, miR-375, miR-143, miR-1, and miR-145.

By MiRWalk 2.0-validated-target miRNA-gene retrieval system, we got 4475 candidate genes of the 26 significant DEMs. The intersection number of these candidate genes and the upregulated DEGs was 633. For the downregulated DEGs, 261 genes were intersected with the 4475 candidate genes. Therefore, the 633 upregulated and the 261 downregulated genes were identified as the final sets of significant DEGs.

### 3.2. GO and Pathway Enrichment

The GO ontology contains three terms: cellular component (CC), molecular function (MF), and biological process (BP). The results demonstrated that the most significant GO terms for upregulated significant DEGs were “protein binding (MF)”, “nucleoplasm (CC)”, and “poly(A) RNA binding (MF)”, whereas for the downregulated significant DEGs, “extracellular matrix (CC)”, “cell surface (CC)”, and “protein binding (MF)” turned to be important (Table 2).

Furthermore, the KEGG pathway analysis indicated that “prostate cancer”, “TGF-beta signaling pathway”, “TNF signaling pathway”, and “focal adhesion” pathways played an essential role in PCa pathogenesis (Table 2).

### 3.3. PPI Network

**Figure 2:**
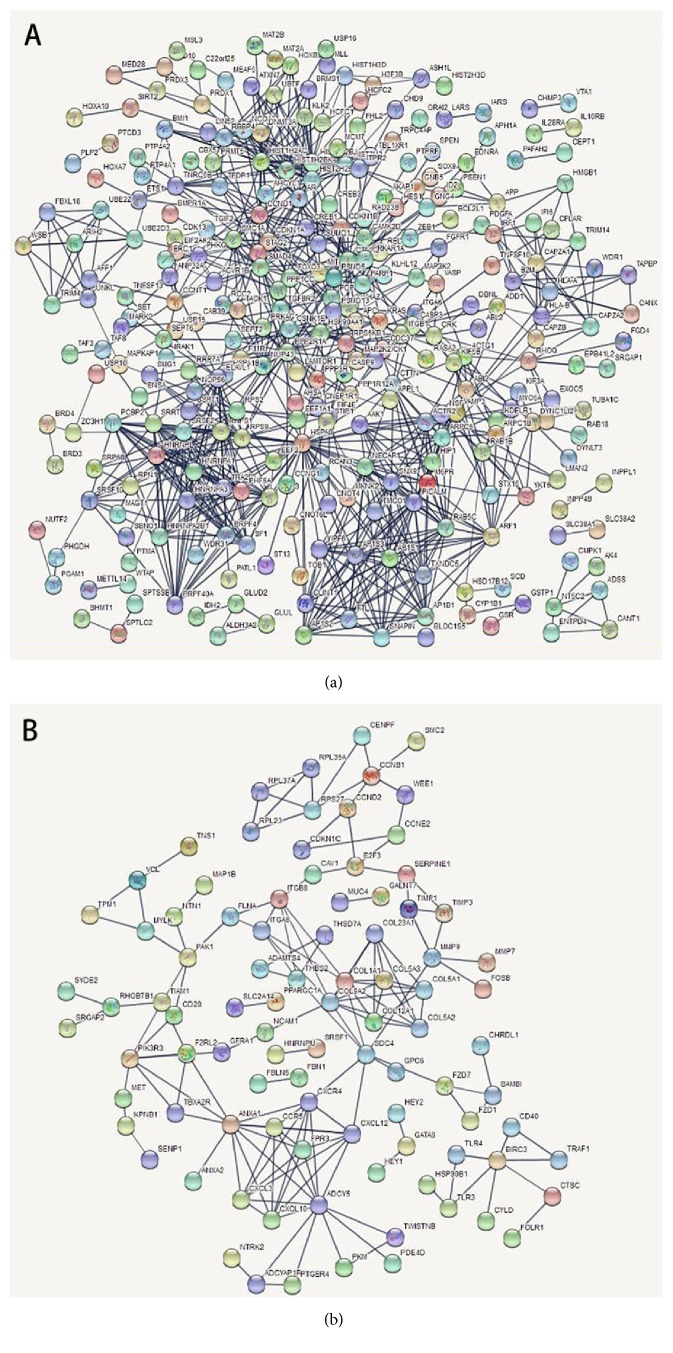
PPI network of DEGs. (a) Upregulated DEGs and (b) downregulated DEGs.

PPI networks were established separately by significant upregulated DEGs (Figure 2(a)) and significant downregulated DEGs (Figure 2(b)). In respect of the former, 294 nodes and 926 edges in total constituted the PPI network. In regard to the latter, the network was composed of 93 nodes and 153 edges.

In a PPI network, the more edges a gene has, the more important role it plays (like a seed). We used the parameter “degree” to calculate edge counts of every single gene in a PPI network. The top 5% degree genes were listed in Table 3, which were assessed as hub genes.

### 3.4. MiRNA-mRNA Regulatory Network

**Figure 3:**
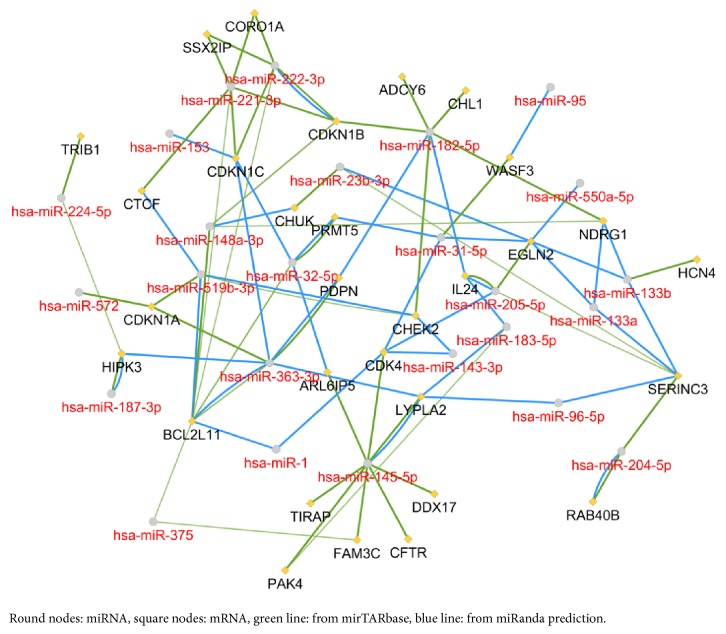
The miRNA-mRNA regulatory network.

As demonstrated in Figure 3, the target genes were predicted by miRanda and miRTarbase, among which two or more miRNA might target the same mRNA. For instance, miR-1, miR-145, miR-143, miR-205, and miR-31 all had interactions with cyclin dependent kinase 4 (CDK4). There were 21 mRNA nodes which might interact with more than two miRNAs.

### 3.5. Validation and Kaplan-Meier Curves

After comparing the expression level of miRNAs in the TCGA dataset, we found that 23 among the 26 significant DEMs were in accordance with the results in GEO databases. However, the expression of miR-1, miR-519b, and miR-572 was not significantly different between PCa samples and normal control prostate tissues. The relationship of miRNAs between the clinical TNM stages was also tested in the TCGA dataset using chi-square analysis. The results of the significant DEMs were shown in Table 4.

**Figure 4:**
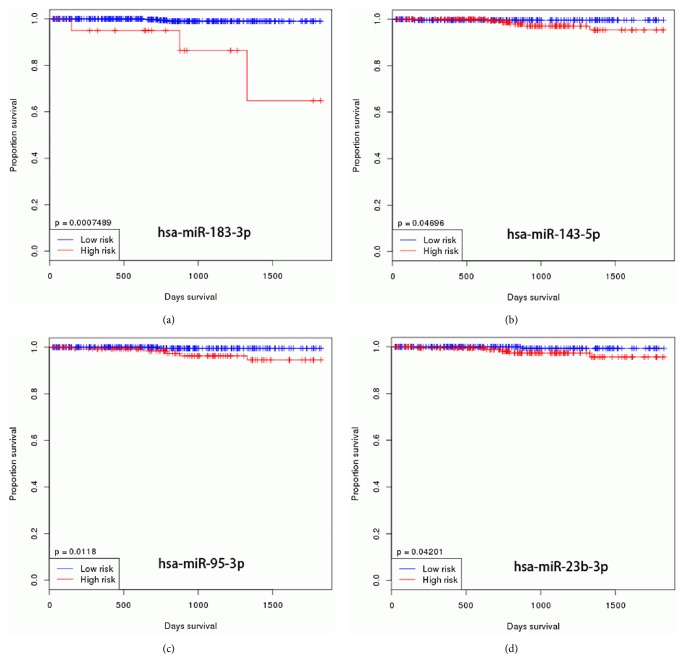
The Kaplan-Meier plots of patients from TCGA data.

We subsequently drew Kaplan-Meier plots (Figure 4) based on the TCGA survival data. Four significant DEMs, miR-95, miR-23b, miR-143, and miR-183 were found related to overall survival (OS) in PCa patients. The higher expression of these miRNAs meant poor OS in patients with PCa.

## 4. Discussion

In the present study, we identified 26 significant DEMs, 633 upregulated DEGs, and 261 downregulated DEGs. The results of functional enrichment analysis indicated that the significant DEGs were related to TGF-beta signaling pathway and TNF signaling pathway in PCa. The key DEGs such as HSPA8, PPP2R1A, CTNNB1, ADCY5, ANXA1, and COL9A2 were found as hub genes in PPI networks. Importantly, some of the DEMs were validated and found correlated with tumor stages and survival, which meant the miRNAs could not only regulate cellular process but also be of high value in clinical practice.

In fact, prostate cancer shares a plenty of pathways with other cancers such as TNF, TGF-beta pathways. Some unique signal pathways like the androgen receptor (AR) and transmembrane protease serine 2 (TMPRSS2) relevant pathways also play a crucial role in PCa. In these complicated processes, miRNAs nearly take part in all key cellular pathways considering that one miRNA can interact with many mRNAs and one mRNA can also interact with many miRNAs. Interestingly, miR-183 was the only one significant DEM expressed in all four different datasets. We assumed that ethnics and sample size may cause this heterogeneity. In accordance with our result, miR-183 has been testified overexpression in PCa serum, tissue, and cell line [16]. Ueno reported that miR-183 could target Dkk-3 and SAMAD4 and has an oncogenic biological behavior [17]. Due to the paralogous property of miR-183, miR-96, and miR-182, these three miRNAs have been studied as miR-183 cluster, which could regulate zinc levels and carcinogenic pathways in prostate cells [18]. MiR-96 was also reported enhancing PCa cell proliferation through FOXO1 [19], which could be speculated in the PPI network (Figure 2(a)). Except for the previously reported miRNAs, there are also some miRNAs not reported in PCa before like miR-95. We found that higher miR-95 expression indicated poor survival based on 494 patients in TCGA. However, we do not know if miR-95 is related to the survival of patients from other regions or countries. The mechanisms and in-depth pathways of miR-95 should also be investigated by experiments in the future.

By constructing the PPI network, we can recognize the key genes that miRNAs may interact with. Since the genes were filtered by the 26 significant DEMs potential targets, there were still 633 upregulated and 261 downregulated. Therefore, when it comes to all the 197 DEMs, the enormous and complicated miRNA-mRNA regulatory network can be imaginable. The hub genes of a network are always important like “seeds”, which connect different signal pathways. In the present study, we identified several hub genes. The regulating mechanisms HSPA8 were reported in malignancies like glioblastoma and myeloid leukaemia [20, 21]. The PPP2R1A mutation in uterine cancer was reported acting through a dominant-negative mechanism to promote cancer cell growth [22]. Besides, emerging evidence points out that the histone cluster is contributed to various kinds of cancers [23]. As a phospholipid-dependent, membrane-binding protein, the ANXA1 upregulation can enhance drug-resistance of PCa therapy [24]. In our predicted miRNA-mRNA network, the key mRNAs like CDKN1A,B,C and CDK4 are cell-cycle related modulators [25]. To sum up, the miRNA regulatory network may provide potential targets for new drug development and treatment. By exploiting the bioinformatic analysis on miRNAs and regulatory pathways, we can discover important and novel potential targets in the tumor-genesis, diagnosis, prognosis, and key mechanisms in different cancers such as lung cancer [26], gastric cancer [27], colorectal cancer [28], and thyroid cancer [29].

Although this study firstly investigated miRNAs' regulatory role in PCa by integrating multiple microarray datasets, we still need to clarify some limitations. Firstly, the stages and aggressiveness of PCa were not restricted. We only researched the PCa versus normal prostate. Key miRNAs and genes in different periods like metastatic castration-resistant PCa call for deeper exploration. Secondly, the source of microarray is only tissues. Body fluid like serum, urine, and prostatic fluid may contain circulating miRNAs, which are more likely to be accepted for clinical application. Thirdly, though we validated our results in TCGA data, the microarray results were not validated by experiments like qRT-PCR, functional experiments in vitro. Further studies are needed to validate our results by experiments on large samples.

## 5. Conclusions

We identified 26 miRNAs that may take part in key pathways like TGF-beta, TNF pathways in prostate cancer regulatory networks. MicroRNAs like miR-23b, miR-95, miR-143, and miR-183 can be utilized in assisting the diagnosis and prognosis of prostate cancer as biomarkers. Further experimental studies are required to validate our results.

## Figures and Tables

**Table 1:** Characteristics of the microarray datasets from GEO database.

Accession/ID	PMID	Platform	Number of prostate cancer tissues	Number of normal prostate tissues (control)	Gene/microRNA
GSE26367	24713434	GPL11350	n=173	n=11	microRNA
GSE64333	26089375	GPL8227	n=27	n=27	microRNA
GSE21036	20579941	GPL8227	n=113	n=28	microRNA
GSE54516	25485837	GPL18234	n=51	n=48	microRNA
GSE55945	19737960	GPL570	n=13	n=8	Gene
GSE60329	27384993	GPL14450	n=54	n=14	Gene
GSE103512	29133367	GPL13158	n=60	n=7	Gene

**Table 2:** Results of top 10 gene ontology (GO) categories and the top 5 Kyoto Encyclopedia of Genes and Genomes (KEGG) pathway analysis.

Category	Term	Description	Gene counts	*P*-value
**Up-regulated gene**				
GO:0005515	MF	protein binding	416	1.40E-20
GO:0005654	CC	nucleoplasm	164	5.20E-14
GO:0044822	MF	poly(A) RNA binding	88	1.80E-12
GO:0005829	CC	cytosol	180	3.00E-12
GO:0005913	CC	cell-cell adherens junction	40	3.20E-12
GO:0098641	MF	cadherin binding involved in cell-cell adhesion	37	4.00E-11
GO:0070062	CC	extracellular exosome	153	2.50E-10
GO:0005634	CC	nucleus	252	3.10E-10
GO:0098609	BP	cell-cell adhesion	33	1.40E-09
GO:0005737	CC	cytoplasm	237	1.90E-08
hsa05215	KEGG	Prostate cancer	14	4.10E-05
hsa04350	KEGG	TGF-beta signaling pathway	11	1.80E-03
hsa05202	KEGG	Transcriptional misregulation in cancer	16	2.80E-03
hsa05210	KEGG	Colorectal cancer	9	3.00E-03
hsa04910	KEGG	Insulin signaling pathway	14	3.30E-03
**Down-regulated gene**				
GO:0031012	CC	extracellular matrix	23	2.20E-10
GO:0009986	CC	cell surface	28	1.30E-08
GO:0005515	MF	protein binding	170	1.00E-07
GO:0005925	CC	focal adhesion	22	1.70E-07
GO:0030198	BP	extracellular matrix organization	16	2.30E-07
GO:0005578	CC	proteinaceous extracellular matrix	17	1.30E-06
GO:0007155	BP	cell adhesion	22	4.80E-06
GO:0045944	BP	positive regulation of transcription from RNA polymerase II promoter	34	8.30E-06
GO:0008201	MF	heparin binding	12	2.40E-05
GO:0030574	BP	collagen catabolic process	8	4.30E-05
hsa04668	KEGG	TNF signaling pathway	12	3.30E-06
hsa04510	KEGG	Focal adhesion	16	4.80E-06
hsa05205	KEGG	Proteoglycans in cancer	15	1.60E-05
hsa05144	KEGG	Malaria	7	2.50E-04
hsa04064	KEGG	NF-kappa B signaling pathway	8	1.00E-03

BP: biological process; CC: cellular component; MF: molecular function

**Table 3:** The top 5% hub genes in the protein-protein interaction (PPI) networks.

Entrez Gene ID	Gene symbol	Full gene name	Degree
**Up-regulated gene**			
3312	HSPA8	heat shock protein family A (Hsp70) member 8	40
5518	PPP2R1A	protein phosphatase 2 scaffold subunit Aalpha	32
1499	CTNNB1	catenin beta 1	25
8334	HIST1H2AC	histone cluster 1 H2A family member c	24
10921	RNPS1	RNA binding protein with serine rich domain 1	23
85236	HIST1H2BK	histone cluster 1 H2B family member k	22
8970	HIST1H2BJ	histone cluster 1 H2B family member j	22
5878	RAB5C	RAB5C, member RAS oncogene family	22
595	CCND1	cyclin D1	21
8301	PICALM	phosphatidylinositol binding clathrin assembly protein	21
25977	NECAP1	NECAP endocytosis associated 1	21
51429	SNX9	sorting nexin 9	21
8349	HIST2H2BE	histone cluster 2 H2B family member e	20
1026	CDKN1A	cyclin dependent kinase inhibitor 1A	20
**Down-regulated gene**			
111	ADCY5	adenylate cyclase 5	12
301	ANXA1	annexin A1	11
1298	COL9A2	collagen type IX alpha 2 chain	10
1277	COL1A1	collagen type I alpha 1 chain	9

**Table 4:** Results of the significant DEMs expression validation and the relationship with PCa clinical stages in TCGA data.

MiRNA ID	Up-regulated in	*P*-value (t-test)	Clinical stages	*P*-value (chi-square)
hsa-miR-133a-3p	normal	8.93E-03	pathologic T/ N status	9.50e-09/ 1.32e-04
hsa-miR-133b	normal	9.53E-07	pathologic T/ N status	7.96e-07/ 8.83e-05
hsa-miR-143-3p	normal	1.10E-13	clinical T/ M status	5.04e-03/3.70e-03
hsa-miR-145-3p	normal	1.65E-07	pathologic T/ N status	7.86e-07/ 1.49e-04
hsa-miR-148a-3p	tumor	7.67E-13	-	-
hsa-miR-148a-5p	tumor	4.66E-10	pathologic T status	2.10e-03
hsa-miR-153-3p	tumor	4.12E-20	pathologic T status	4.30e-02
hsa-miR-182-5p	tumor	7.57E-16	clinical T/ M status	4.23e-03/ 3.06e-02
hsa-miR-183-5p	tumor	1.56E-15	pathologic T status/ Clinical M Status	7.85e-03/ 1.25e-03
hsa-miR-187-3p	normal	1.52E-10	-	-
hsa-miR-204-5p	normal	2.62E-06	pathologic T/ N status	2.44e-03/ 1.77e-02
hsa-miR-205-5p	normal	1.39E-02	pathologic T status	1.17e-02
hsa-miR-221-3p	normal	6.18E-10	pathologic T/ N status	4.10e-07/ 3.70e-04
hsa-miR-221-5p	normal	1.44E-05	pathologic T/ N status	5.69e-03/ 5.19e-03
hsa-miR-222-3p	normal	5.76E-07	pathologic T/ N status	4.71e-09/ 2.77e-04
hsa-miR-222-5p	normal	1.17E-04	pathologic T status/ Clinical M Status	1.37e-03/ 4.81e-02
hsa-miR-224-5p	normal	2.31E-02	pathologic T status	5.62e-04
hsa-miR-23b-3p	normal	8.14E-03	pathologic T/ N status	9.60e-03/ 3.24e-02
hsa-miR-31-3p	normal	3.97E-02	-	-
hsa-miR-32-3p	tumor	2.96E-03	pathologic T/ N status	3.29e-03/ 3.81e-02
hsa-miR-363-3p	tumor	1.18E-08	pathologic N status	2.92e-02
hsa-miR-375	tumor	8.87E-13	-	-
hsa-miR-550a-5p	tumor	1.08E-04	-	-
hsa-miR-7-5p	tumor	1.70E-03	pathologic T/ N status	4.28e-02/ 2.03e-03
hsa-miR-95-3p	tumor	8.29E-06	pathologic T status	7.17e-03
hsa-miR-96-5p	tumor	1.65E-15	pathologic N status/ Clinical M Status	4.72e-02/ 5.81e-03

## Data Availability

All raw data in this article can be obtained by emailing the corresponding author.
